# Nicotinamide and NAFLD: Is There Nothing New Under the Sun?

**DOI:** 10.3390/metabo9090180

**Published:** 2019-09-10

**Authors:** Maria Guarino, Jean-François Dufour

**Affiliations:** 1 Hepatology, Department for BioMedical Research, University of Bern, 3008 Bern, Switzerland; 2 Gastroenterology, Department of Clinical Medicine and Surgery, University of Naples Federico II, 80131 Naples, Italy; 3 University Clinic of Visceral Surgery and Medicine, Inselspital Bern, 3008 Bern, Switzerland

**Keywords:** nicotinamide, NAFLD, steatosis

## Abstract

Nicotinamide adenine dinucleotide (NAD) has a critical role in cellular metabolism and energy homeostasis. Its importance has been established early with the discovery of NAD’s therapeutic role for pellagra. This review addresses some of the recent findings on NAD physiopathology and their effects on nonalcoholic fatty liver disease (NAFLD) pathogenesis, which need to be considered in the search for a better therapeutic approach. Reduced NAD concentrations contribute to the dysmetabolic imbalance and consequently to the pathogenesis of NAFLD. In this perspective, the dietary supplementation or the pharmacological modulation of NAD levels appear to be an attractive strategy. These reviewed studies open the doors to growing interest in NAD metabolism for NAFLD diagnosis, prevention, and treatment. Future rigorous clinical studies in humans will be necessary to validate these preliminary but promising results.

## 1. Introduction

The global diabesity (diabetes and obesity) [1] epidemic has dramatically increased the prevalence of nonalcoholic fatty liver disease (NAFLD), such that it is the most frequent cause of chronic liver disease. NAFLD is considered to be the liver manifestation of the metabolic syndrome, because of its frequent association with dyslipidemia, cardiovascular disease, obstructive sleep apnea, vitamin D deficiency, and other components of the metabolic syndrome, and insulin resistance is central to its pathogenesis [2,3].

Liver steatosis is the hallmark histologic feature of NAFLD, and it is the result of triglyceride accumulation in the hepatocytes cytoplasm. Liver lipid accumulation arises from an imbalance between lipid accumulation and removal, which is linked to increased liver lipogenesis, increased lipid uptake, and/or reduced triglyceride export or β-oxidation [4,5]. Liver secretion of triglycerides as very low-density lipoprotein (VLDL) particles for delivery to peripheral tissues is a crucial pathway for the mobilization of hepatic fat. Defects in VLDL processing are directly linked to hepatic steatosis. Jiang et al. showed that non-alcoholic steatohepatitis (NASH) was related to an increment in VLDL particle size, while hepatic fibrosis was related to a reduction in the concentration of small VLDL particles [6]. Moreover, there is a relationship between choline deficiency and accumulation of liver lipid, which is why choline-deficient diets are often used to induce NAFLD in animal models. Within hepatocytes, choline may be oxidized for phosphatidylcholine synthesis. Liver phosphatidylcholine is used to build the monolayers of VLDL, and its deficiency increases de novo hepatic lipogenesis [7].

The present model for NAFLD pathophysiology, called “the multiple-hit hypothesis”, defines NAFLD as the manifestation of environmental and genetic factors, including the dysfunction of different organs and organelles, together with the intricate interaction between hepatocytes and other cells (such as stellate cells and Kupffer) in the liver [8]. Additionally, the liver is a hub for several metabolic pathways defining NAFLD as a multistep, progressive systemic disease.

## 2. NAD: Behind Its Metabolism

**Figure 1:**
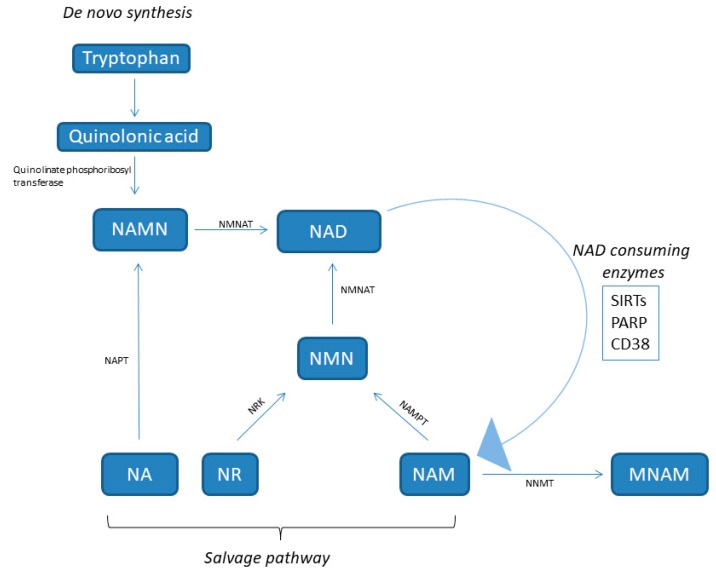
NAD synthesis pathways. NA, nicotinic acid; NAD, nicotinamide adenine dinucleotide; NAM, nicotinamide; NAMN, nicotinic acid mononucleotide; NAPT, nicotinic acid phosphoribosyltransferase; NMN, nicotinamide mononucleotide; NMNAT, nicotinamide nucleotide adenylyltransferase; NR, nicotinamide riboside; NRK, NR kinase; NNMT, nicotinamide-N-methyltransferase; PARP, poly (ADP ribose) polymerase; NNMT, nicotinamide N-methyltransferase; NAMPT, nicotinamide phosphoribosyltransferase; SIRT, sirtuin.

Nicotinamide adenine dinucleotide (NAD) is a hydride acceptor producing the reduced NADH, as well as the derivate phosphorylated dinucleotide pair NADP/NADPH, which is required for many cellular biosynthetic pathways and for protecting cells from reactive oxygen species (ROS). The keystone function of NAD is to facilitate hydrogen transfer in metabolic pathways as enzyme cofactors dealing with hydrogen transfer in reductive or oxidative metabolic reaction. So, it plays a central role in basic energy metabolism such as assisting with mitochondrial electron transport, glycolysis, the oxidation of fatty acids and amino acids in mitochondria, and the citric acid cycle. NAD is also a substrate for signaling enzymes such as poly (ADP ribose) polymerase (PARP), sirtuins (SIRTs), and ADP ribosyl transferases, called “NAD consumers” [9] (Figure 1). For example, it is involved in repairing and maintaining genomic integrity, thanks to PARP, which transfers ADP-ribose from NAD to itself, histones, and other proteins at sites of DNA damage.

The cellular NAD pool is created by a balance between the activity of NAD-consuming and synthesizing enzymes [10,11,12]. NAD concentrations display the cell energy state and are modulated by physiological processes. In fact, during fasting, caloric restriction, and exercise, NAD levels increase. Conversely, caloric excess and aging diminish NAD levels [13]. 

NAD is synthesized from four distinct biosynthetic precursors in two different pathways (Figure 1). De novo synthesis (the deamidated pathway) uses as precursor the dietary amino acid tryptophan, which is metabolized to create biosynthetic intermediates. In particular, the creation of unstable α-amino-β-carboxymuconate-ε-semialdehyde (ACMS) forms a branching point of the deaminated pathway. The ACMS is subjected to both non-enzymatic cyclization or complete enzymatic oxidation to quinolinic acid, and this is the first limiting step [14]. The second limiting mechanism involves the catalytic conversion of quinolinic acid to nicotinic acid mononucleotide (NAMN) by quinolinate phosphoribosyl transferase. Next, NAMN is transformed into NAD by the nicotinamide mononucleotide adenylyltransferase (NMNAT) enzyme. This pathway is recognized as the minor contributor to the total NAD pool [14]. 

Dietary vitamin B3 compounds, including nicotinic acid (NA), also known as niacin, NAM, and nicotinamide riboside (NR), supply as NAD biosynthetic precursors and are rescued from the diet (the amidated pathway) for generating cellular NAD. This salvage pathway is the most relevant for NAD homeostasis [15]. NA is converted to NAMN by nicotinic acid phosphoribosyltransferase (NAPT), which is afterward converted to NAD by NMNAT. The NAM and NR are transformed into NMN by nicotinamide phosphoribosyltransferase (NAMPT) and NR kinase (NRK) enzymes, respectively. Finally, NMN is enzymatically transformed into NAD by NMNAT [15].

NAMPT, also known with the name visfatin, is a highly conserved protein with cytokine functions, which is expressed in almost all tissues and cells (Figure 1) [16]. In particular, it is an essential regulator of the intracellular NAD pool by catalyzing the formation of nicotinamide mononucleotide (NMN) from nicotinamide and 5′-phosphoribosyl-1-pyrophosphate, which is the limiting step in the NAD salvage pathway [17]. NAMPT has both intracellular and extracellular forms in mammals. The extracellular NAMPT (eNAMPT) is secreted from adipocytes [18], hepatocytes [19], and leucocytes [20] and circulates in the blood where, additionally to its enzymatic function, it has also cytokine-like actions [16,21,22]. In virtue of its NAD biosynthetic activity, intracellular NAMPT (iNAMPT) controls the activity of NAD-dependent and consuming enzymes, such as SIRTs [23], the NADase CD38 (a cyclic ADP-ribose synthesis) [24], and PARPs [25], by which it controls mitochondrial biogenesis, cellular metabolism [26], and adaptive responses to oxidative, inflammatory, genotoxic, and proteotoxic stress [27]. Genotoxic stress and nutrient deprivation activate NAMPT, which protects cells from these stresses through the maintenance of the mitochondrial NAD level [23].

The NAD levels are also regulated by the cytosolic enzyme nicotinamide-N-methyltransferase (NNMT), which methylates nicotinamide to produce N1-methyl nicotinamide (MNAM) toward the universal methyl donor S-adenosylmethionine as a methyl donor (Figure 1). NNMT is mainly expressed in the liver, but also in other organs such as muscle, adipose tissue, and heart. An increase of NNMT expression has been observed in obesity and diabetes [28,29,30]. 

SIRTs are NAD-dependent deacylases [31]. SIRTs have key roles in response to environmental and nutritional perturbations, such as DNA damage, oxidative stress, and fasting. For this reason, SIRTs have to be considered as nutritional sensors that operate in regulating glucose and lipid homeostasis, inflammatory responses, and cell death [23,32,33,34]. Additionally, SIRTs influence cells’ metabolism through the regulation of the circadian clock machinery with the deacetylation of central clock components in the liver [35,36]. Accordingly, NAD synthesis is controlled by the circadian machinery to furnish a crucial link from the clock oscillator to metabolic pathways [37]. NAD is synthesized with circadian oscillations, leading to a circadian schedule of SIRT activation and mitochondrial metabolism, such as the oxidation of fatty acids [38]. SIRTs’ activity is dependent on its cofactor NAD and it is sensitive to the cellular NAD levels [39], designating NAD as a rate-limiting substrate for their reactions [32,40,41]. As NAM is the product of SIRT-catalyzed deacetylation reactions, high levels of NAM have been used as a SIRTs inhibitor [42]. This drives speculation that enzymes involved in NAD synthesis could control SIRTs’ activity. For example, an increment in NAD was proposed by Lin et al. to mediate the health span and extension of life by dietary restriction [43], and recently, studies demonstrated that the activity of SIRTs declines with aging by a systemic reduction in NAD levels [44,45]. 

## 3. NAD Involvement in NAFLD Pathogenesis

In the last years, an emerging role of NAD metabolism in protection against NAFLD stimulated a growing interest. Von Shönfels et al. performed a small-molecule metabolite screen of human hepatic tissue to find metabolic markers related to NASH histology. According to its concentration in liver tissue, they suggested a protective effect of NA, which was subsequently verified in a nutritional animal model of NAFLD showing a marked effect on steatosis and transaminases levels with NA supplementation [46]. NAD deficiency decreases the oxidation of fatty acids, promoting steatosis [47]. Usually, the triglycerides are broken down into glycerol and fatty acids, so they can enter into the mitochondria and proceed on with fatty acid oxidation. Fatty acids shift in this pathway as Coenzyme A (CoA) derivatives utilizing NAD. The acetyl groups created by the β-oxidation of the fatty acid take part in the activity of the Krebs cycle, causing the formation of NADH. The reduced coenzyme (NADH) is oxidized by leaving the protons and electrons to oxygen in the mitochondria to synthesize ATP in the electron transport system [48]. So, NAD deficiency causes a reduction of β-oxidation, and consequently the accumulation of triglycerides in the hepatocytes (steatosis).

The control of rate-limiting enzymes of NAD biosynthesis avoids the negative effects of high-fat diet (HFD) and keeps up insulin sensitivity and glucose homeostasis. Penke et al. [49] reported increased hepatic NAD levels in mice under HFD thanks to increased NAMPT expression. So, it seems that NAD deficiency is a crucial risk factor for NAFLD resulting from having compromised the NAMPT-controlled NAD salvage pathway in liver [50]. Plasma levels of eNAMPT may be closely linked to NAFLD, obesity, diabetes, and atherosclerosis [51,52,53,54]. Moreover, decreased NAMPT expression in NAMPT +/− mice, which reduced circulating NMN levels and decreased NAD levels in brown adipose tissue, impaired glucose-stimulated insulin secretion [22]. This event can be rescued by NMN supplementation, suggesting that the maintenance of NAD concentrations is critical for pancreatic function [22]. 

**Figure 2:**
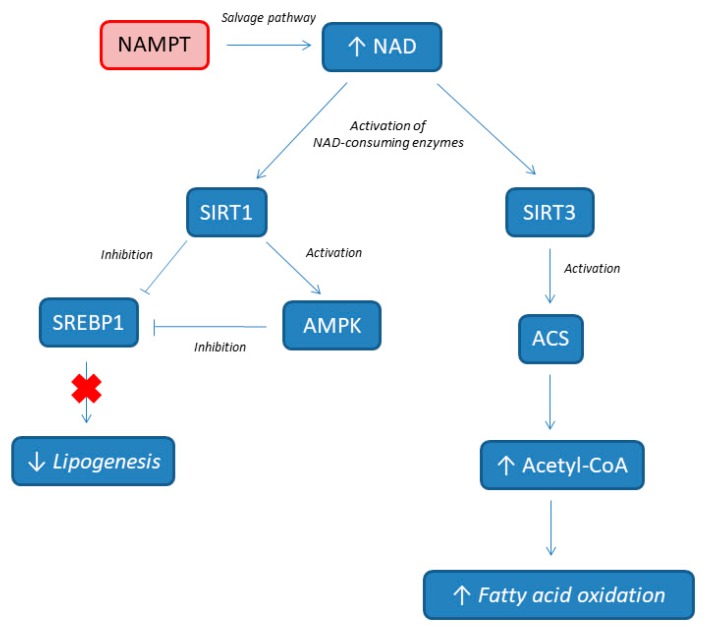
NAMPT involvement in lipids metabolism. NAMPT, nicotinamide phosphoribosyltransferase; NAD, nicotinamide adenine dinucleotide; ACS, acetyl-CoA synthetase; AMPK, AMP-activated protein kinase; SIRT, sirtuin; SREBP1, sterol regulatory element-binding protein 1.

The mechanisms of NAMPT protecting the liver from HFD are depicted in Figure 2. NAMPT induces the production of NAD by activating the NAD salvage pathway, and consecutively, the augmented NAD (as a substrate) activates the SIRT 1 and 3 signaling pathways, alleviating HFD-induced hepatic steatosis. De novo lipogenesis (DNL) is known to be high in individuals with NAFLD, and provides about 26% of hepatic lipids [55,56]. The NAMPT is critical for the formation of acetyl-CoA and for the increase of fatty acid oxidation by providing NAD for SIRT3 with the activation of acetyl-CoA synthetase (ACS) [57]. At the same time, the activation of SIRT1 by NAMPT promotes the deacetylation of sterol regulatory element-binding protein 1 (SREBP1), which inhibits SREBP1 activity, resulting in the lower expression of lipogenesis genes, including fatty acid synthase (FAS) and acetyl-CoA carboxylase (ACC). Additionally, SIRT 1 directly activates AMP-activated protein kinase (AMPK), which further inhibits SREBP1 activity. All together, these results show that NAMPT modulates processes involved in NAFLD pathogenesis (such as de novo lipogenesis and fatty acid oxidation). Accordingly, Zhou et al. showed that dominant negative-NAMPT transgenic mice, under normal chow, display systemic NAD decrease and had a moderate NASH phenotype, with enhanced oxidative stress, lipid accumulation, impaired insulin sensitivity, and triggered inflammation in liver. These features deteriorate further under HFD [50]. 

NNMT has also been associated to the development of diabetes, obesity, and metabolic syndrome [28,29,30]. An increase of NNMT expression has been observed in obesity and diabetes [28,29,30], probably because NNMT controls lipid, cholesterol, and glucose metabolism by stabilizing SIRTs [58]. In humans, adipose tissue NNMT expression and its product MNAM correlate positively with insulin resistance. Kannt et al. [29] showed an increased expression of NNMT in the adipose tissue of diabetic patients according to the insulin resistance severity, suggesting that NNMT could be a “bad actor” limiting fuel oxidation and promoting fat storage. NNMT protein levels are upregulated in the liver and adipose tissue of mouse models of insulin resistance and obesity, and NNMT knockdown has a protective effect against the metabolic consequences of HFD [28], suggesting that NNMT may have a critical role in NAFLD pathogenesis. The dietary regulation of liver NNMT expression, the site of its major expression, shows some interesting patterns. The ketogenic diet suppresses liver NNMT expression, contributing to the increased liver and serum cholesterol levels in this model [59]. Conversely, caloric restriction increased NNMT liver expression, promoting SIRT1 protein stability, which mediates several metabolic effects of caloric restriction [60]. Liver NNMT expression inversely correlates with serum triglycerides (TGs), cholesterol, and free fatty acid levels, suggesting that increased liver NNMT expression is associated with a better metabolic profile, contrary to its expression in adipose tissue [28,29]. Furthermore, a genome-wide association study showed significant associations between the risk of developing NASH and a specific single-nucleotide polymorphisms (SNPs) in the NNMT gene (rs694539) [61]: in this case, subjects with the AA genotype showed a statistically significant increased NASH risk, while the GG genotype seemed to be protective. Similarly, Hasan et al. showed that the AA genotype correlates with the degree of steatosis as detected by the controlled attenuation parameter, even if it does not correlate with the degree of fibrosis detected by FibroScan [62].

## 4. NAD as Biomarker for NAFLD Diagnosis

The identification of non-invasive biomarkers has become a major focus of interest in NAFLD. Since the diagnosis of NASH is still a histological one, the dramatic increase in the prevalence of NAFLD and its severity spectrum mean that liver biopsy is not feasible for all patients. Current plasma biomarkers include predictive models for diagnosing or grading steatosis (such as the fatty liver index) or staging fibrosis (such as the NAFLD fibrosis score), and other ones specific to NAFLD (such as the BARD and NAFLD fibrosis scores), even if some have been initially developed in a hepatitis C setting (AST/ALT ratio, APRI, FIB-4) [63].

Several studies evaluated the relationship between NAD metabolism and NAFLD [29,64,65,66,67,68] (Table 1). Human studies investigated how plasma and liver NAMPT protein levels are affected in subjects with steatosis and NAFLD [64,65,66,67,68]. Gaddipati et al. [64] showed that a significant reduction in the NAMPT levels of the visceral adipose tissue is associated to degree of steatosis in NAFLD patients. Similarly, Amirkalali et al. [68] showed that higher serum NAMPT is associated with lower liver DNL in female subjects (probably associated with a higher adipose tissue DNL according to the higher fat mass), while the only significant association in male subjects was between serum NAMPT and liver fat content, probably for the inflammatory role of NAMPT. Thus, the plasma NAMPT levels could have a different meaning for each sex because of the opposing effects of liver and adipose tissue DNL on NAFLD pathogenesis. Conversely, Kannt et al. [29] showed that NNMT mRNA in adipose tissue and 1-methylnicotinamide serum concentrations are higher in patients with insulin resistance and correlate with insulin resistance severity. An additional interesting result is that improvements of insulin sensitivity obtained with exercise and bariatric surgery are associated with a reduction of NNMT expression in adipose tissue and of 1-methylnicotinamide serum levels [29]. 

## 5. NAD Supplementation for NAFLD Prevention

The evidence for using dietary supplementation to prevent chronic disease is a longstanding issue of debate. Several evidences are emerging to support the hypothesis that supplementation with NAD precursors could protect against metabolic imbalance and liver steatosis (Table 2) [12,49,69,70,71]. A supplementation study with NMN showed its property to restore NAD levels either in nuclear and mitochondrial cells compartments and to prevent diet-induced and age-induced diabetes in C57BL/6 mice [12]. Tao et al. showed that NAMPT gives resistance to hepatic steatosis through NAD synthesis [69], and NR supplementation gives protection against steatosis in mice under high-fat/high-sucrose diet [70,71]. NAM supplementation protects hepatocytes from palmitate-induced cell death, and autophagy induction contributes to the anti-lipotoxic property of NAM through SIRT1 activation in hepatocytes. Additionally, NAM prevents hepatic alterations in glucose-6-phosphate dehydrogenase and the redox state, and attenuates increased serum FFA, oxidative stress, inflammation, and hepatic damage in high fructose or high glucose consumption-induced liver steatosis in rats [72]. Lastly, Komatsu et al. showed that NNMT and NAM supplementation causes liver steatosis and fibrosis, although increased lipid metabolism and decreased adiposity. NNMT overexpression induces genes for liver steatosis and fibrosis by decreasing tissue NAD content and methylation pool, suggesting that NNMT connects NAD and methionine metabolism and causes NAFLD progression [73]. Thus, NAD supplementation may represent a preventive treatment for metabolic dysfunctions such as diabetes, and NAFLD spectrum disease, from steatosis to NASH.

## 6. NAD Supplementation for NAFLD Treatment

The relevance of dietary NAD precursors in health is well known, thanks to the historical use of NA and NAM in the treatment of dietary tryptophan deficits (pellagra) and hyperlipidemia, although high-dose NA use is limited by painful flushing, while high-dose NAM is hepatotoxic [74,75]. In fact, the use of NA is associated with a flush of face and chest and a sensation of warmth or burning. NA causes flushing principally by releasing prostaglandins D2 and E2 from skin cells, which afterwards dilates skin arterioles [76,77]. The precursors NA, NMN, and NR, but also PARP or CD38 inhibitors, rise NAD levels in different mice cells and tissues [12,13,70]. Boosting NAD concentrations can be therapeutic in metabolic diseases such as diabetes [12,53] and NAFLD [70], and potentially protects against obesity [51] and age-related disorders (Table 3).

Due to its ability to increase NAD synthesis without inducing side effects [44,70], NR has been used in mice to increase NAD metabolism and improve health in models of metabolic stress, showing that NR abolishes DNA damage in HFD-fed mice [70,78]. Canto et al. [70] treated mice with NR (400 mg/kg animal weight per day), demonstrating an increase of NAD levels in muscle and liver. Mice under HFD were protected from body weight increase and showed an improvement of mitochondrial function and fatty acids oxidation as a fuel source. In accordance with increases in tissue NAD levels, SIRT1 and SIRT3 were upregulated [70]. NR also ameliorated insulin sensitivity in weight-matched mice [70]. Similarly, Zhou et al. [50] demonstrated that the oral administration of NR corrects NAFLD phenotypes induced by NAD deficiency alone or combined with HFD. Trammell et al. [79] performed a clinical study enrolling 12 healthy subjects receiving three single doses of NR, demonstrating that NR supplementation safely induces NAD metabolism at all doses. They also demonstrated that NR is more orally bioavailable than NAM, which is more orally bioavailable than NA. The capability of NR to increase ADPR is threefold higher than NAM. This validates NR as the preferred NAD precursor vitamin for boosting NAD and NAD-consuming activities in liver. No dose-dependent side effects of NR have been reported, contrary to high-dose NAM, which may lead to liver damage [15]. Shi et al. [80] carried out a dose–response dietary intervention mice study using a wide range of NR (from 5 to 900 mg NR per kg of an obesogenic diet), concluding that 30 mg/kg diet constitutes the best concentration to reinforce metabolic health. These studies showed the powerful biological effects of NR in mitigating the negative consequences of HFDs [70,71,81,82], suggesting that NAD substrates supplementation may be a promising therapeutic strategy for preventing and treating NAFLD/NASH. 

Another possibility to modulate NAD levels consists of using NMN. Supplementation with NMN, an enzymatic product of NAMPT, improves diabetes [12,13] and other damages such as vascular dysfunction, oxidative stress [83], and cognitive impairment [84]. Yoshino et al. demonstrated that increasing NAD biosynthesis by the intraperitoneal injection of NMN improves glucose homeostasis in obese mice, and that NAMPT activity is altered by HFD and can cause diabetes [12]. Similarly, supplementation with MNAM significantly reduces hepatic cholesterol and triglycerides concentrations, by suppressing fatty acid and cholesterol synthesis and the expression of lipogenic and cholesterol synthesis genes [58]. MNAM supplementation produces a selective reduction in larger lipoprotein particles but not high-density lipoprotein, suggesting that MNAM or its derivatives could be used to reduce low-density lipoprotein levels [58].

Another attractive angle to modulate NAD levels consists in targeting the activity of NAD-consuming enzymes, such as CD38 [10] and PARPs [11]. Several studies showed that CD38 knockout (KO) mice have higher NAD levels than Wild-type (WT) animals, and are protected against obesity and metabolic syndrome [10,85]. The treatment of obese mice with CD38 inhibitors augments intracellular NAD concentrations and improves glucose and lipid homeostasis [86]. Increased PARP activity causes an elevated consumption of cellular NAD, which is associated to increased ATP consumption, compromising energy balance and facilitating cell death [87]. Upon persistent PARP activation, decreased mitochondrial ATP production inhibits NAD re-synthesis, creating a feed-forward loop in ATP-consuming processes, and resulting in metabolic catastrophe and cell death. PARP inhibition causes an increase in NAD levels. Rucaparib (a PARP inhibitor) significantly increases hepatic NAD levels, as previously described with NAM treatment [88], while in PARP1 KO liver, NAD levels were similar to those in treated PARP1 WT liver. So, CD38 and PARP inhibition combined with NAD precursors may be an intriguing therapeutic perspective for NAFLD [13].

Finally, Katsiuba et al. presented an additional mechanism for increasing NAD levels toward the inhibition of the ACMS decarboxylase with a selective inhibitor recently developed, TES-991. ACMS decarboxylase inhibition in a mouse model of diet-induced NAFLD increased levels of NAD and the activation of SIRT1 with improvement of the NAFLD phenotype, without systemic side effects [14].

## 7. Conclusions

Until now, there is still no approved drug for the treatment of NAFLD, and although lifestyle modification appears beneficial in patients with NAFLD, no single approach is likely to be suitable for all patients. NAD reduction might be caused by the imbalance in NAD biosynthesis and depletion, both of which occur in NAFLD. NAD reduction may induce NAFLD through decreased SIRT activities in the nucleus and mitochondria. The supplementation of key NAD intermediates, such as NMN and NR, can ameliorate NAFLD.

## Figures and Tables

**Table 1:** NAD as biomarker for NAFLD diagnosis.

Biomarker	Study Design	Analyzed Tissue	Results	Ref.
NAMPT	77 NAFLD patients vs. 38 control patients (all undergoing diagnostic laparoscopy)	Visceral adipose tissue (VAT)	Reduction of NAMPT levels in VAT according to the degree of steatosis	Gaddipati et al. [64]
NAMPT	69 obese women with NAFLD vs. 19 obese women vs. 38 healthy women	Liver tissue and serum	Serum NAMPT and its liver expression are higher in obese women with NAFLD, irrespective of the presence of diabetes	Auguet et al. [65]
NAMPT	58 NAFLD patients vs. 27 healthy controls	Liver tissue and serum	NAFLD patients had decreased NAMPT expression both in serum and in liver tissue, with no difference between simple steatosis and NASH	Dahl et al. [52]
NAMPT	40 severely obese patients with NAFLD	Liver tissue	Positive association between NAMPT expression and the fibrosis stage in NAFLD	Kukla et al. [67]
NAMPT	62 NAFLD patients (32 males, 30 females)	Serum	Higher serum NAMPT in women was associated with a lower hepatic DNL index, while in men, it was associated with higher hepatic fat, and had no association with the DNL index	Amirkalali et al. [68]
NNMT and 1-Methylnicotinamide	199 patients undergoing abdominal surgery (111 diabetic and 88 non-diabetic); 60 individuals on a 12-week exercise program (20 diabetic, 20 insulin-resistant, and 20 with normal glucose tolerance)	Serum and white adipose tissue (WAT)	Patients with diabetes have a twofold higher NNMT expression. There is an inverse correlation between insulin sensitivity and plasma 1-methylnicotinamide and WAT NNMT expression.	Kannt et al. [29]

NAMPT, nicotinamide phosphoribosyltransferase; NNMT, nicotinamide-N-methyltransferase.

**Table 2:** NAD supplementation for NAFLD prevention.

Preventive Supplementation	Study Design	Results	Ref.
NMN	C57BL/6, HFD vs. control diet	NMN ameliorates glucose intolerance by restoring NAD levels, enhances hepatic insulin sensitivity, and restores gene expression related to oxidative stress, inflammatory response, and circadian rhythm, partly through SIRT1 activation.	Yoshino et al. [12]
Nicotinamide	HepG2 cells and alpha mouse liver (AML)-12 hepatocyte transfected with human SIRT1 siRNA under palmitate-elicited hepatotoxicity	Nicotinamide supplementation protects hepatocytes against palmitate-induced cell death. SIRT1 inhibition abrogates the nicotinamide anti-lipotoxic effect.	Shen et al. [42]
NR	C57Bl/6J, HFD vs. control diet; murine C2C12 myoblasts, murine Hepa1.6, and human HEK293T cells, with or without deletion of the SIRT3 gene	NR prevents diet-induced obesity by enhancing energy expenditure, reducing cholesterol levels, and increasing intracellular and mitochondrial NAD content both in cell and in vivo experiments. NR enhances SIRT1 and SIRT3 activity and energy expenditure, and ameliorates the oxidative performance of skeletal muscle and brown adipose tissue.	Canto et al. [70]
NR	C57BL/6J mice, high-fat and high-sucrose diet vs. control diet; primary hepatocytes from SIRT1 floxed or SIRT3 floxed mice	NR prevents NAFLD by inducing a sirtuin-dependent mitochondrial unfolded protein response, triggering an adaptive mitohormetic pathway to increase hepatic β-oxidation and mitochondrial complex content and activity.	Gariani et al. [71]
NAM	Male Sprague–Dawley rats were randomly distributed into six groups according to the following treatments: (1) Control; (2) Glucose; (3) Glucose+NAM 0.06%; (4) Glucose+NAM 0.12%; (5) Fructose; and (6) Fructose+NAM 0.12%.	NAM attenuates increases in levels of FFA, thiobarbituric acid reactive substances, and markers of hepatic damage induced by high glucose or fructose. NAM decreases hepatic steatosis. NAM only partially prevented changes in the glutathione/glutathione disulfide levels and redox potential, as well as pro-inflammatory conditions. NAM mitigates increases in hepatic glucose-6-phosphate dehydrogenase mRNA, protein levels, and specific activity induced by glucose or fructose.	Mejia et al. [72]
NAM	C57Bl/6J transgenic mice overexpressing NNMT vs. wild type, HFD + water containing 1% NAM	NNMT overactivation decreases the NAD content in the liver and decreases gene activity related to fatty acid oxidation by inhibiting SIRT3 and fibrosis by reducing the tissue NAD content and methylation pool.	Komatsu et al. [73]

NR, nicotinamide riboside; NMN, nicotinamide mononucleotide; HFD, high-fat diet.

**Table 3:** NAD supplementation for NAFLD treatment.

Treatment	Study design	Results	ref
NR	C57Bl/6J, HFD vs. control diet.	Long-term NR administration in vivo lowers HFD-induced body weight gain by enhancing energy expenditure, and ameliorates insulin-sensitivity and cholesterol profiles.	Canto et al. [70]
NR	Dominant negative (DN)-NAMPT transgenic C57BL/6J, HFD vs. control diet.	DN-NAMPT mice under control diet displays systemic NAD reduction and had moderate NAFLD phenotypes, including lipid accumulation, enhanced oxidative stress, triggered inflammation, and impaired insulin sensitivity in liver. All these NAFLD phenotypes deteriorate further under HFD challenge. Oral administration of NR completely corrects these NAFLD phenotypes induced by NAD deficiency alone or with HFD.	Zhou et al. [50]
NR	C57BL/6JRcc mice, semi-synthetic obesogenic diet containing 0.14% l-tryptophan and either 5, 15, 30, 180, or 900 mg NR per kg diet	There is a dose–response effect to NR; in particular, mice fed a 30 mg NR/kg diet are more metabolically flexible than the wide range of other NR concentrations. Moreover, in epididymal white adipose tissue, the gene expression of Peroxisome-proliferator-activated receptor- γ (Ppar- γ), Superoxide dismutase-2 (SOD2) and Peroxiredoxin 3 (Prdx3) - are significantly upregulated in mice fed 30 mg NR/kg.	Shi et al. [80]
NR	Obese-diabetic KK/HlJ mice, control or NR group	Total cholesterol concentration in the liver, glucose control, and levels of serum insulin and adiponectin are improved by NR. At liver histology, NR rescues the disrupted cellular integrity of the mitochondria and nucleus of obese–diabetic KK mice. In addition, NR treatment significantly improves hepatic pro-inflammatory markers, including tumor necrosis factor-alpha, Interleukin (IL) 6, and IL-1. These results demonstrate that NR attenuates hepatic metaflammation by modulating the NLRP3 inflammasome.	Lee et al. [81]
NR	C57BL/6J, HFD vs. control diet	NR improves glucose tolerance, and reduces weight gain, liver damage, and hepatic steatosis.	Trammell et al. [82]
MNAM	C57BL/6J, HFD vs. control diet	MNAM significantly lowers liver and serum cholesterol and TG levels, while also suppressing fatty acid and cholesterol synthesis and the expression of lipogenic and cholesterol synthesis genes. MNAM-supplemented mice have higher liver SIRT1 protein expression. Consistent with higher SIRT1 protein expression, liver FoxO1 acetylation is significantly lower. MNAM-fed mice had significantly lower liver expression of the pro-inflammatory cytokines.	Hong et al. [58]
Flavonoid Apigenin (CD38 inihibitor)	C57BL/6, HFD vs. control diet	Apigenin inhibits CD38 and is associated with increased NAD and decreased protein acetylation, likely through the activation of SIRT1. Apigenin improves glucose homeostasis in vivo and promotes fatty acid oxidation in the liver.	Escande et al. [86]
PARP-1 inhibitors	HeLa cells exposed to the PARP-1-activating agent N-methyl-N’-nitro-N-nitrosoguanidine (MNNG) or to PARP-1 inhibitors after MNNG exposure.	PARP-1 hyperactivity in the nucleus rapidly impairs ATP production in mitochondria, whereas the release of the pro-apoptotic factors AIF/Cyt-c from mitochondria only occurs several hours after PARP-1 hyperactivation. PARP-1 inhibitors are able to prevent MNNG-induced nucleotide depletion, apoptosis-inducing factor (AIF) release, and cell death.	Cipriani et al. [87]
Rucaparib (PARP1 inhibitor)	PARP1 wild-type (WT) and PARP1 knock-out (KO) mice	In PARP1 WT livers, the NAD concentration in the rucaparib-treated group was significantly higher when compared with the concentration in untreated mice, and similar to the concentration in KO mice.	Almeida et al. [88]
TES-991 (ACMS decarboxylase inhibitor)	C57BL/6J under methionine-choline deficient (MCD) diet	Supplementing the MCD diet with TES-991 increases hepatic NAD, attenuates hepatic steatosis and plasma transaminases levels, protects against hepatic lipid accumulation, attenuates inflammation, recovers hepatic SOD2 activity and ATP content, and reverses NAFLD changes in the transcription of genes involved in ROS defense, β-oxidation, inflammation, and mitochondrial function.	Katsyuba et al. [14]

NR, nicotinamide riboside; MNAM, N1-methyl nicotinamide; PARP1, poly (ADP ribose) polymerase 1; HFD, high fat diet.

## Abbreviations

ACC, acetyl-CoA carboxylase; ACMS, amino-β-carboxymuconate-ε-semialdehyde; ACS, acetyl-CoA synthetase; ADPR, ADP ribose; AMPK, AMP-activated protein kinase; DNL, de novo lipogenesis; eNAMPT, extracellular NAMPT; FAS, fatty acid synthase; FFA, free fatty acid; HCC, hepatocellular carcinoma; HFD, high fat diet; iNAMPT, intracellular NAMPT; KO, knock-out; MNAM, N1-methyl nicotinamide; NA, nicotinic acid; NAD, nicotinamide adenine dinucleotide; NADP, phosphorylated nicotinamide adenine dinucleotide; NAFLD, nonalcoholic fatty liver disease; NAM, nicotinamide; NAMN, NA mononucleotide; NAMPT, nicotinamide phosphoribosyltransferase; NAPRT1, nicotinate phosphoribosyltransferase domain containing 1; NASH, non-alcoholic steatohepatitis; NMN, nicotinamide mononucleotide; NMNAT, nicotinamide nucleotide adenylyltransferase 3; NNMT, nicotinamide-N-methyltransferase; NR, nicotinamide riboside; NRK, NR kinase; PARP, poly (ADP ribose) polymerase; ROS, reactive oxygen species; SIRT, sirtuin; SREBP1, sterol regulatory element-binding protein 1; TNF-α, tumor necrosis factor-α; WT, wild-type.
